# Reliability and Validity of the Self- and Interviewer-Administered Versions of the Global Physical Activity Questionnaire (GPAQ)

**DOI:** 10.1371/journal.pone.0136944

**Published:** 2015-09-01

**Authors:** Anne H. Y. Chu, Sheryl H. X. Ng, David Koh, Falk Müller-Riemenschneider

**Affiliations:** 1 Saw Swee Hock School of Public Health, National University of Singapore, Singapore, Singapore; 2 PAPRSB Institute of Health Sciences, Universiti Brunei Darussalam, Jalan Tungku Link, Gadong, Brunei Darussalam; 3 Institute of Social Medicine, Epidemiology and Health Economics, Charité University Medical Centre Berlin, Berlin, Germany; University Of São Paulo, BRAZIL

## Abstract

**Objective:**

The Global Physical Activity Questionnaire (GPAQ) was originally designed to be interviewer-administered by the World Health Organization in assessing physical activity. The main aim of this study was to compare the psychometric properties of a self-administered GPAQ with the original interviewer-administered approach. Additionally, this study explored whether using different accelerometry-based physical activity bout definitions might affect the questionnaire’s validity.

**Methods:**

A total of 110 participants were recruited and randomly allocated to an interviewer- (n = 56) or a self-administered (n = 54) group for test-retest reliability, of which 108 participants who met the wear time criteria were included in the validity study. Reliability was assessed by administration of questionnaires twice with a one-week interval. Criterion validity was assessed by comparing against seven-day accelerometer measures. Two definitions for accelerometry-data scoring were employed: (1) total-min of activity, and (2) 10-min bout.

**Results:**

Participants had similar baseline characteristics in both administration groups and no significant difference was found between the two formats in terms of validity (correlations between the GPAQ and accelerometer). For validity, the GPAQ demonstrated fair-to-moderate correlations for moderate-to-vigorous physical activity (MVPA) for self-administration (*r*_s_ = 0.30) and interviewer-administration (*r*_s_ = 0.46). Findings were similar when considering 10-min activity bouts in the accelerometer analysis for MVPA (*r*_s_ = 0.29 vs. 0.42 for self vs. interviewer). Within each mode of administration, the strongest correlations were observed for vigorous-intensity activity. However, Bland-Altman plots illustrated bias toward overestimation for higher levels of MVPA, vigorous- and moderate-intensity activities, and underestimation for lower levels of these measures. Reliability for MVPA revealed moderate correlations (*r*_s_ = 0.61 vs. 0.63 for self vs. interviewer).

**Conclusions:**

Our findings showed comparability between both self- and interviewer-administration modes of the GPAQ. The GPAQ in general but especially the self-administered version may offer a relatively inexpensive method for measuring physical activity of various types and at different domains. However, there may be bias in the GPAQ measurements depending on the overall physical activity. It is advisable to incorporate accelerometers in future studies, particularly when measuring different intensities of physical activity.

## Introduction

A continuous expanding body of literature shows that physical activity is amongst the most important determinants in the development of chronic diseases such as diabetes, stroke, hypertension, obesity, and coronary heart disease [1,2]. Along with the worldwide public health attention placed on this issue, there is compelling evidence from systematic reviews suggesting a dose-response relationship between low levels of physical activity and increased all-cause mortality [3,4]. The World Health Organization (WHO) guidelines stated that in order to stay healthy and improve health, adults aged 18–64 years should perform at least 150-minute of moderate-intensity aerobic physical activity or at least 75-minute of vigorous-intensity aerobic physical activity throughout the week, with each aerobic activity performed in bouts of at least 10-minute duration [5].

Questionnaires and objective tools (e.g. accelerometers and pedometers) are the most commonly used instruments in assessing physical activity. Of all the measuring methods, questionnaires are most widely used in large-scale epidemiological studies owing to their relatively low cost, minimal burden on participants and higher applicability of use [6]. One of the most commonly used questionnaires is the Global Physical Activity Questionnaire (GPAQ), developed by the WHO for the WHO STEPwise Approach to Chronic Disease Risk Factor Surveillance [7]. In addition to subjective measures, objective tools have gained increasing widespread use for quantifying physical activity. However, one of the major limitations associated with objective tools is the inability to distinguish between different domain-specific activities (work, transportation and recreational activities). Therefore, the complementary roles between questionnaires and objective tools have equal widespread applications in the physical activity research field. While accelerometers are considerably more costly, they currently reflect the state of the art measurement tool for the objective assessment of physical activity and sedentary behavior in population-based studies [8]. Comparison of questionnaires with accelerometers is therefore a standard approach to determine the criterion validity of physical activity questionnaires.

The GPAQ was initially developed for face-to-face interviews conducted by trained interviewers. To date, there are two studies which have tested the GPAQ using only the self-administration rather than interviewer-administration [9,10]. In relation to interviewer-administration, the self-administered questionnaires have the logistical advantages of saving cost especially when utilizing postal mail or online questionnaires [11], and also eliminating interviewer bias [12]. On the other hand, the feasibility of using self-administered questionnaires for population-based physical activity assessment could be hampered by respondent bias, especially among participants with reading problems [12]. There have been studies performed on the validity of the GPAQ which demonstrated low-to-moderate correlations of the physical activity scores against a pedometer (*r* = 0.31 to 0.54) and an accelerometer (*r* = 0.20 to 0.34), with reliability ranging from correlations of *r* = 0.39 to 0.81 [10,13–15]. However, no comparison between self-administrations and interviews was carried out. A more important issue in this case would be to achieve comparability and consistency between self- and interviewer administered questionnaires.

The aim of this study was therefore to psychometrically compare the self-administered and original interviewer-administered versions of the GPAQ among an English-speaking adult population in Singapore. Additionally, given that the GPAQ asks questions on activity performed in accumulated bouts of at least 10-minute at one time, the second aim of this study further explored the validity in two scenarios: (1) Considering total-minute of activity, and (2) applying the definition of a sustained 10-minute bout on top of the total definition to determine physical activity measures from the accelerometer data.

## Materials and Methods

### Study design and participant selection

This was a cross-sectional study. A convenience sample of 113 working adults and students from different faculties and departments of a large public University and a university hospital in Singapore was recruited between February 2014 and June 2014. Participants were invited to join this study through printed posters or via a mass email advertisement sent through the university’s internal mail. Individuals who indicated interest were approached. Study inclusion criteria were:

1. Men or women aged 21 and older
2. Working adults or students
3. Singapore citizens or permanent residents
4. Of three predominant ethnic groups (Chinese, Malay, and Indian)
5. Absence of physical disabilities or illness that would restrict normal activities
6. English-literate.

The study was approved by the National University of Singapore Institutional Review Board (NUS-IRB Ref No.: B-14-021).

### Sample size calculation

Sample size estimation was estimated using the Power and Sample Size (PS) Program. Referring to a previous study [15] which assessed the criterion validity of the GPAQ against the accelerometers, a Spearman correlation, *r*_s_ = 0.40 was assumed for detecting a statistically significant coefficient. To achieve a power of 80% with the level of significance at 0.05, the required sample size was 50. Considering the investigation of both self-administered and interviewer-administered versions of the GPAQ, the total sample size to be enrolled was 100 participants.

### Procedure

The goals and procedures of the study were explained to each participant and written informed consent was obtained from everyone before the study began. Participants’ gender, age, education level, ethnicity, height and weight information were self-reported. Each person in the sample was randomly assigned using a computer generated random list to one of the two administration modes: (1) self-administered; or (2) interviewer-administered. Participants were contacted and scheduled to hand in the accelerometers and the log sheet, followed by completion of the retest questionnaire (constituting the reliability testing component). The mean time interval between the first and second questionnaire administrations was 7 days.

### Global Physical Activity Questionnaire (GPAQ)

The GPAQ (both self- and interviewer-administered versions) comprises of 16 items that quantify the physical activity levels of a normal active week for the participants. The WHO developed the GPAQ to estimate the total weekly volume of MVPA, moderate- and vigorous-intensity activities in a typical week among these three domains: work, transportation and recreational activities. Particularly, household activity was included in the work domain. The GPAQ data with invalid or missing values were cleaned and processed using the GPAQ analysis guide (WHO, 2012). The duration and frequency of physical activity (min/day) participation in three domains (activity at work, travel to and from places, and recreational activities) over a typical week were recorded. Activities are classified into three intensity levels: vigorous (8 metabolic equivalent task; METs), moderate (4 METs) and inactivity (1 MET) [5]. A summary estimate of total MVPA in min/day was calculated by combining the activity score of both moderate- and vigorous-intensity activity for each work and recreational activity domain. Participants were further classified into three activity intensity categories (low-, moderate-, or high-intensity activity level) according to their total physical activity per week (MET-minute per week) based on the GPAQ guidelines with the following criteria:

- High: A person meeting any of the following criteria is classified in this category: (1) Vigorous-intensity activity on at least three days achieving a minimum of 1500 MET-minute per week or seven or more days of any combination of walking, moderate- or (2) vigorous-intensity activities achieving a minimum of 3000 MET-minute per week.
- Moderate: A person not meeting the criteria for the ‘high’ category, but meeting any of the following criteria is classified in this category: (1) Three or more days of vigorous-intensity activity of at least 20-minute per day or five or more days of moderate-intensity activity or (2) walking for at least 30-minute per day or five or more days of any combination of walking, moderate- or vigorous-intensity activities achieving a minimum of 600 MET-minute per week.
- Low: A person not meeting any of the above-mentioned criteria [5].

According to the GPAQ guidelines, participants could also be classified into two activity groups to reflect whether they are meeting weekly physical activity recommendations:

- Sufficiently active: Participants engaged in at least (1) 30-minute of moderate-intensity activity or walking per day on at least five days of a typical week; or (2) 20-minute of vigorous-intensity activity per day on at least three days of a typical week; or (3) 5 days of any combination of walking and moderate- or vigorous-intensity activities achieving a minimum of at least 600 MET-minute per week.
- Inactive: Those who did not meet one the above-mentioned criteria.

### Actigraph (wGT3X-BT) accelerometer

The Actigraph wGT3X-BT monitor (ActiGraph, LLC, Pensacola, Florida, USA) is a triaxial accelerometer (4.6 cm x 3.3 cm x 1.5 cm, with a weight of 19 grams) worn on the waist using an elastic belt to secure above the right hip bone for measuring the amount and frequency of human movements. The monitor was initialized at a sample rate of 30Hz to record activities for free-living conditions. Participants were instructed to wear the waist-worn accelerometer 24-hour/day for seven consecutive days. However, they were permitted to remove the accelerometers if they feel uncomfortable wearing the device during sleep. They were advised to remove the accelerometers only during water-based activities such as bathing or immersing the body in water. They were required to complete a daily log sheet (indicating start/stop date) while maintaining their normal activities during the study period. Instruction manual on the proper usage of accelerometers was given to each participant for additional guidance. Data were downloaded and integrated into 60-s epochs.

### Actigraph Sleep and Wear Time Validation

Accelerometry-derived physical activity data were summarized based on two definitions: (1) total-minute of activity and, (2) accumulation of activity in bouts of least 10-minute. To determine wear time for the accelerometers, log sheets filled out by participants were used to provide a reference point of start and stop dates.

For the treatment of the 24-hour accelerometry data, a recently published automated algorithm by Tudor-Locke and Barreira et al. was adapted and slightly modified for the detection of nocturnal sleep in waist worn accelerometry to better reflect adult sleep time in the present study [16,17]. The previous algorithm was built upon a widely evaluated sleep algorithm for classification of each epoch into sleep and wake states [18]. Sleep onset was defined as the first of five consecutive minutes scored as sleep, and to identify nocturnal sleep periods, only sleep onset between 9pm to 6am the following morning was included for analysis. Sleep offset was set as the first of 10 consecutive minutes of awakening time, following sleep onset. If sleep offset lies between 11pm to 5am, extension of time is needed to mark sleep offset by an additional 10-min (total 20-min). The sleep onset/offset index was excluded if length of sleep period was <160-min. Adjacent sleep periods were combined if there was a lapse of <20-min between them. As some participants did not wear the device during sleep, the wear time validation algorithm was applied to distinguish the sleep period from invalid wear time for these individuals.

After identifying sleep time, the remaining waking minutes were cleaned by application of a separate non-wear algorithm to identify valid wear time during waking hours. The algorithm was set to use: 1) Zero value threshold of activity counts (ActiGraph) during a nonwear time interval, 2) 90-minute of time window for consecutive minutes of zero counts, and the artefactual movements detection was set to allow interruptions of 2-minute interval or less with the upstream or downstream 90-minute consecutive zero-count window [19]. Participants with a wear time corresponding to at least 10 hours during waking time per day (i.e., ≥600 total wear min/day), collected over four full days or more were included in analysis.

Freedson’s cut points for triaxial accelerometers were used to determine time spent in moderate-intensity activity (2691–6166 counts per minute [CPM]), and vigorous-intensity activity (>6167 CPM) [20]. Accelerometer values were divided by the number of valid wear days to obtain the average number of minutes per day.

Accelerometry data were downloaded using ActiLife software (Version 6) and time spent in various physical activity levels (min/day) was assessed using the accelerometry package in R (Version 3.1.3).

### Statistical Analysis

Descriptive sociodemographic characteristics, physical activity estimates, and categorization of participants into various physical activity levels were presented for all participants and separately for each mode of administration as median and interquartile range (IQR) or number (%). The differences in participants’ characteristics and accelerometry-based summary estimates of physical activity between the two modes of administration were assessed by a Fisher's exact test (if cells have an expected frequency of five or less) or a chi square test for categorical variables, and Mann-Whitney U test for continuous variables.

Reliability and criterion validity of the GPAQ was assessed as follows:

1. Test-retest reliability between the GPAQ for each mode of self- and interviewer-administration (min/day) at each activity intensity level (MVPA, moderate- and vigorous-intensity physical activity) and by physical activity domains (work, transport and recreational activity domains). Reliability testing was assessed using Spearman’s rank correlation test and the two-way mixed model (single measure) intraclass correlation coefficient (ICC) with 95% confidence interval (CI). ICCs were interpreted as follows: values below 0.40 are considered as poor agreement, 0.40–0.59 as moderate agreement, 0.60–0.79 as good agreement and ≥0.80 as excellent agreement [21]. Weighted Cohen’s Kappa statistic was used to assess the reliability of the GPAQ in categorizing individuals whether or not they meet the physical activity guidelines. Happ Landis and Koch’s guide for interpreting agreement for categorical data was utilized: ≤0.20 slight, 0.21–0.40 fair, 0.41–0.60 moderate, 0.61–0.80 substantial, >0.80 almost perfect [21].
2. Criterion validity between the GPAQ at follow-up and accelerometry-derived data for each activity intensity level (MVPA, moderate- and vigorous-intensity physical activity). The validity of each group was assessed using Spearman’s rank correlation test and Bland-Altman plots with the 95% limits of agreement (LOA). Bland-Altman plots were used to assess the agreement of physical activity at different activity intensities (min/day) between the GPAQ and accelerometer. To examine the variation of the Bland-Altman plots across different modes of administration, sensitivity analyses by self- and interviewer-administered groups were conducted.

Thereafter, to assess if the Spearman correlations and Kappa values differed significantly between the self- and interviewer administered groups, a Z-test was used on the difference in the values [22,23].

All statistical procedures were performed using SPSS software (Statistical Package for the Social Sciences, Chicago, IL, version 22) and Stata statistical software (StataCorp LP, College Station, Texas, version 13). Significance level was set at 0.05.

## Results

**Fig 1:**
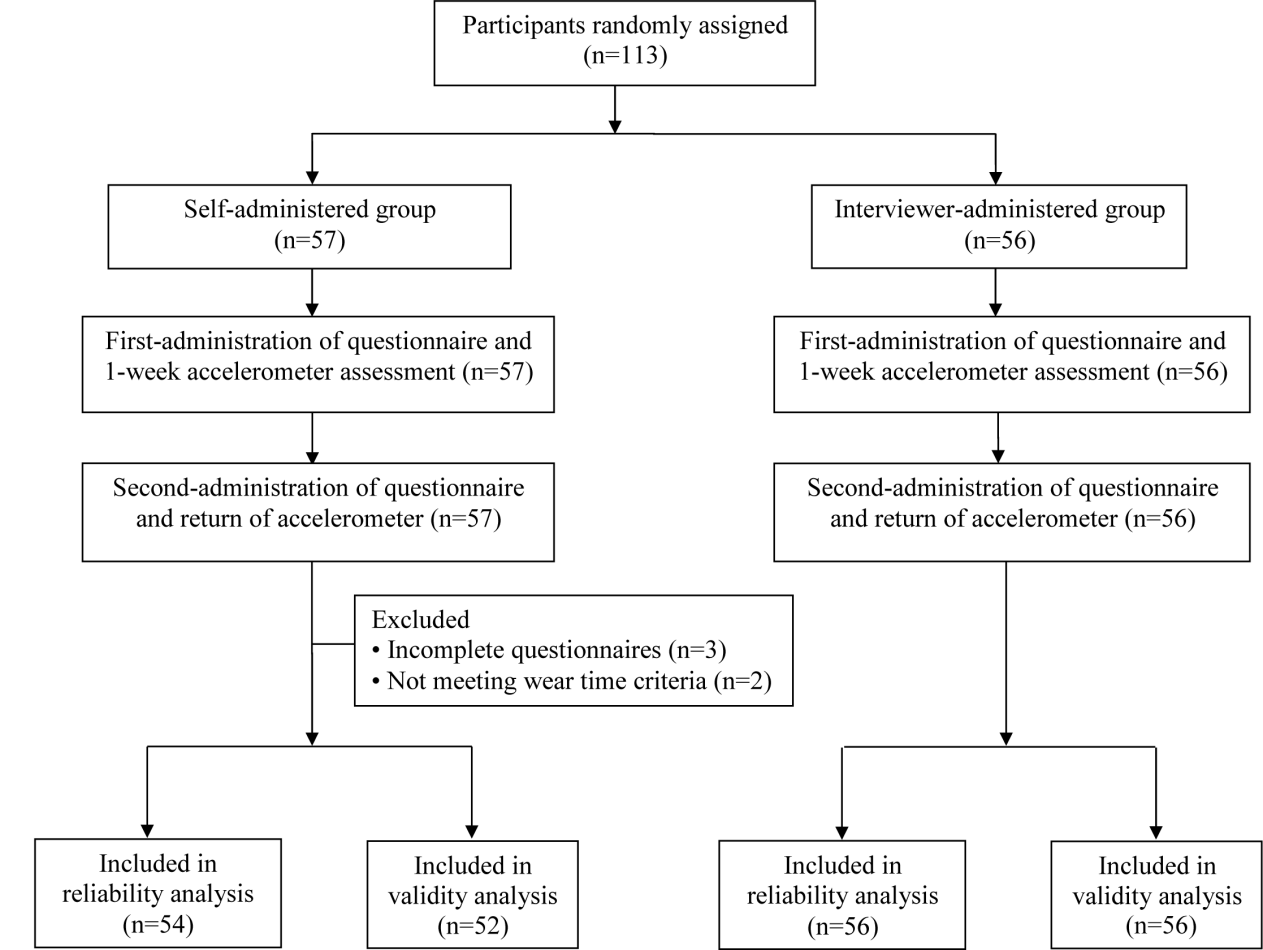
Flowchart of participants’ recruitment.

A total of 110 participants were included in the test-retest reliability study, of which 108 participants who met the wear time criteria were included in the validity study. A flow chart of study participants’ recruitment is shown in Fig 1.

Table 1 shows the sociodemographic characteristics of the total sample and stratified according to the different modes of administration. The majority of the participants from the total sample were full-time working adults (90.0%). Participants were predominantly female (70.9%), relatively young, had a university degree (68.2%), work in the university (66.4%) and of Chinese ethnicity (82.7%). No significant difference in gender, age, educational level, occupation, departments and race was found between the two different modes administration.

**Table 1:** Characteristics comparison of self- vs. interviewer-administered participants.

Characteristics		Self-administered group	Interviewer-administered group	All	p-value^a^
		(n = 54)	(n = 56)	(n = 110)	
		n (%)	n (%)	n (%)	
**Gender**					0.10
Female		34 (63.0)	44 (78.6)	78 (70.9)	
Male		20 (37.0)	12 (21.4)	32 (29.1)	
**Age, median years (IQR)**		32.0 (23.0–67.0)	31.0 (20.0–68.0)	31.0 (26.8–47.3)	0.94
**Education**					0.32
	Secondary	3 (5.6)	6 (10.7)	9 (8.2)	
	Technical school or diploma	11 (20.4)	15 (26.8)	26 (23.6)	
	University	40 (74.0)	35 (62.5)	75 (68.2)	
**Occupation**					0.76
	Staff	48 (88.9)	51 (91.1)	99 (90.0)	
	Student	6 (11.1)	5 (8.9)	11 (10.0)	
**Departments**					0.18
	Public University	40 (74.1)	33 (58.9)	73 (66.4)	
	University Hospital	14 (25.9)	23 (41.1)	37 (33.6)	
**Race**					0.38
	Chinese	44 (81.5)	47 (83.9)	91 (82.7)	
	Malay	3 (5.5)	4 (7.1)	7 (6.4)	
	Indian	7 (13.0)	5 (9.0)	12 (10.9)	

^a^ Test of significance between self- and interviewer-administered groups.

### Self-reported physical activity

Table 2 illustrates the daily minutes of engagement in domain-specific physical activity based on the test and retest of the GPAQ by self- and interviewer-administrations.

**Table 2:** Test-retest reliability of physical activity domains (min/day) by self- and interviewer-administered groups (n = 110).

GPAQ domain (min/day)	Self-administered group (n = 54)				Interview-administered group (n = 56)				All (n = 110 )			
	Test	Retest	*r*_s_ (95% CI)	ICC (95% CI)	Test	Retest	*r*_s_ (95% CI)	ICC (95% CI)	Test	Retest	*r*_s_ (95% CI)	ICC (95% CI)
**MVPA**	80.0 (50.0–146.3)	90.0 (40.0–165.0)	0.63 (0.44–0.77)	0.79^a^ (0.75–0.91)	90.0 (52.8–171.3.0)	70.0 (45.0–127.5)	0.61 (0.41–0.75)	0.28^a^ (0.10–0.48)	90 (51.5–151.3)	82.5 (43.8–142.5)	0.63 (0.50–0.73)	0.54 (0.40–0.66)
**Vigorous: work**	0 (0)	0 (0)	0.71 (0.55–0.82)	0.59 (0.38–0.74)	0 (0)	0 (0)	-^c^	-^c^	0 (0)	0 (0)	0.71 (0.60–0.79)	0.59 (0.45–0.70)
**Vigorous : recreation**	20.0 (0–60.0)	5.0 (0–60.0)	0.86 (0.77–0.92)	0.76 (0.62–0.85)	20.0 (0–60.0)	0 (0–60.0)	0.82 (0.71–0.89)	0.70 (0.54–0.81)	20.0 (0–60.0)	0 (0–60.0)	0.83 (0.76–0.88)	0.73 (0.63–0.81)
**Total vigorous**	20.0 (0–60.0)	5.0 (0–60.0)	0.86 (0.77–0.92)	0.74 (0.59–0.84)	20.0 (0–60.0)	0 (0–60.0)	0.82 (0.71–0.89)	0.70 (0.54–0.81)	20.0 (0–60.0)	0 (0–60.0)	0.83 (0.76–0.88)	0.82 (0.62–0.80)
**Moderate: work**	0 (0–30.0)	0 (0)	0.55 (0.33–0.71)	0.49 (0.26–0.67)	0 (0–26.3)	0 (0–13.8)	0.41 (0.17–0.61)	0.29 (0.04–0.51)	0 (0–30.0)	0 (0–30.0)	0.48 (0.32–0.61)	0.37 (0.20–0.52)
**Moderate: recreation**	15.0 (0–48.8)	10.0 (0–41.3)	0.46 (0.22–0.65)	0.55 (0.33–0.71)	0 (0–38.8)	0 (0–43.8)	0.59 (0.39–0.74)	0.69 (0.52–0.80)	0 (0–41.3)	5.0 (0–41.3)	0.53 (0.38–0.65)	0.60 (0.47–0.71)
**Transport**	20.0 (0–30.0)	20.0 (0–30.0)	0.47^b^ (0.23–0.66)	0.75^a^ (0.60–0.85)	0 (0–30.0)	20.0 (0–30.0)	0.73^b^ (0.58–0.83)	0.26^a^ (0.01–0.48)	20 (0–30.0)	20 (0–30.0)	0.60 (0.47–0.71)	0.47 (0.31–0.60)
**Total moderate**	35.0 (0–61.3)	30.0 (0–90.0)	0.64 (0.45–0.78)	0.62 (0.43–0.76)	20.0 (0–60.0)	30.0 (0–60.0)	0.52 (0.30–0.69)	0.36 (0.11–0.56)	30.0 (0–60.0)	30.0 (0–71.3)	0.58 (0.44–0.69)	0.48 (0.32–0.61)

Median (IQR) of physical activity on average per day.

^a^ Significantly different *r*_*s*_ between self- and interviewer-administered groups.

^b^ Significantly different ICC between self- and interviewer-administered groups.

^c^ Not estimable due to lack of readings for vigorous activity.

### Test-retest reliability

For self-administered group, median daily duration of total MVPA at work, transportation and recreational activities increased from test to retest (Table 2). Conversely, the interviewer-administered group reported decreased test-retest changes of total MVPA. For the assessment of reliability on total MVPA, Spearman’s test revealed moderate correlations (*r*_*s*_ = 0.61 and 0.63 for self- and interviewer-, respectively, p<0.001) (Table 2). Agreement on the reliability assessment of total MVPA was significantly higher for self-administered group than interviewer-administered group (ICC: 0.79 vs 0.28, p<0.001).

Among the activity domains, strongest correlations and agreement were presented for vigorous recreational activities for both groups (*r*_*s*_ = 0.82 and 0.86, p<0.001; ICC: 0.70 and 0.76 for interviewer- and self-administered groups respectively). Moderate to strong correlations and agreement were observed for all domain-specific variables (work, transport and recreation) for self- (*r*_*s*_ = 0.46–0.86, p<0.001; ICC: 0.49–0.85) and interviewer-administered group (*r*_*s*_ = 0.41–0.82, p<0.001; ICC: 0.26–0.70). Within the transport domain, there was a statistically significant difference in the Spearman’s coefficients between the two modes; in which the reliability coefficient in the interviewer-administered group was significantly higher than that of the self-administration (*r*_*s*_ = 0.47 vs. 0.73, p = 0.03). Nevertheless, within the same (transport) domain, a higher agreement was found in the self-administered group as compared to the interviewer- administered group (ICC: 0.75 vs. 0.26, p<0.001).

Within all participants, the Spearman’s coefficients for the reliability of questionnaires were between moderate to excellent (*r*_*s*_ = 0.48–0.83, all p<0.001). The highest correlation was apparent within recreational vigorous activity and total vigorous activity domains (*r*_*s*_ = 0.83, p<0.001 for both domains).

Table 3 shows the proportion of participants classified into categories of physical activity level based on two criteria. The proportions of participants categorized as highly physically active were larger for self-administered group than interviewer-administered group in both test and retest. Most of the participants were classified in the low physical activity level category for both administration groups. The agreement for categorizing participants into low-, moderate- and vigorous- activity levels was fair to moderate for interviewer- (Kappa: 0.33), self-administered group (Kappa: 0.41) and all participants (Kappa: 0.24), respectively.

**Table 3:** Proportion of participants according to the GPAQ classification of meeting recommended physical activity levels (low, moderate, high) by self- and interviewer-administered groups (n = 110).

		Self-administered group (n = 54)		Interview-administered group (n = 56)		All (n = 110)	
		Test	Retest	Test	Retest	Test	Retest
		n (%)	n (%)	n (%)	n (%)	n (%)	n (%)
**GPAQ activity levels** ^a^							
	High	14 (26.0)	12 (22.2)	9 (16.0)	10 (17.9)	23 (20.9)	22 (20.0)
	Moderate	20 (37.0)	19 (35.2)	17 (30.4)	20 (35.7)	37 (33.6)	39 (35.5)
	Low	20 (37.0)	23 (42.6)	30 (53.6)	26 (46.4)	50 (45.5)	49 (44.5)
	Kappa (95% CI)	0.41 (0.21–0.61)		0.33 (0.20–0.47)		0.24 (0.03–0.45)	
**Meeting physical activity recommendations**							
	Sufficiently active	34 (63.0)	30 (55.6)	25 (44.6)	29 (51.8)	59 (53.6)	59 (53.6)
	Inactive	20 (37.0)	24 (44.4)	31 (55.4)	27 (48.2)	51 (46.4)	51 (46.4)
	Kappa (95% CI)	0.35 (0.10–0.60)		0.36 (0.12–0.60)		0.36 (0.19–0.53)	

^a^ Classification according to the GPAQ guidelines.

In the second criteria for categorizing participants as active or inactive, among all participants, 53.6% of participants met the physical activity recommendations for both the test and the retest conditions. The agreement for categorization of participants into meeting sufficient physical activity level was fair for all participants, self- and interviewer-administered groups (Kappa: 0.35–0.36). There was no statistically significant difference when each kappa estimate was compared between the two groups.

### Accelerometry-derived physical activity

Table 4 summarizes estimates of accelerometer measured physical activity in all, self- and interviewer-administered participants. There was no difference in the valid wearing days, wear time and accelerometry-derived physical activity at each intensity level. Generally, vigorous physical activity contributed very little to total MVPA. Assessment of the total-minute of accelerometry-derived physical activity demonstrated 46.5 min/day of MVPA, 0.4 min/day of vigorous-intensity activity and 43.2 min/day of moderate-intensity activity. With a 10-minute bout definition, participants recorded 16.7 min/day of MVPA, 0 min/day of vigorous-intensity activity and 15.1 min/day of moderate-intensity activity.

**Table 4:** Accelerometry-based physical activity summary estimates (median, IQR).

		Self-administered group	Interview-administered group	All	p-value^a^
		(n = 52)	(n = 56)	(n = 108)	
**Valid wearing day (day/week)**		7.0 (6.0–7.0)	7.0 (6.0–7.0)	7.0 (6.0–7.0)	0.20
**Valid wear time** ^b^ **(h/day)**		16.6 (14.8–17.2)	15.9 (14.8–17.4)	16.3 (14.8–17.3)	0.53
**Total-min physical activity (min/day)**					
	MVPA	48.5 (37.3–56.6)	45.7 (29.4–59.8)	46.5 (32.7–56.7)	0.47
	Vigorous	0.4 (0–4.3)	0.4 (0–3.1)	0.4 (0–3.6)	0.57
	Moderate	43.2 (35.1–55.7)	43.5 (28.1–56.7)	43.2 (32.0–55.8)	0.56
**10-min bout physical activity (min/day)**					
	MVPA	17.0 (10.5–31.8)	14.9 (5.9–28.1)	16.7 (7.2–30.3)	0.23
	Vigorous	0 (0–3.5)	0 (0)	0 (0–2.1)	0.30
	Moderate	15.8 (9.9–25.1)	13.3 (5.6–27.5)	15.1 (6.5–25.5)	0.31

^a^ Test of significance of difference between self- and interviewer-administered groups.

^b^ Valid wear time during waking hours.

### Criterion validity

In general, moderate correlations were found between the GPAQ at follow-up and accelerometry-derived estimates at all physical activity intensity levels (Table 5). No significant difference in the correlation coefficients was found between the two modes of administration in terms of their criterion validity. When assessing the overall physical activity (without 10-minute bout definition), the strongest correlations were observed for vigorous-intensity activity. The correlation of vigorous-intensity activity was also higher among interviewer-administered group (*r*_s_ = 0.52, p<0.001) than it was among self-administered group (*r*_s_ = 0.38, p = 0.005), as well as when both groups were assessed together (*r*_s_ = 0.45, p<0.001). There was moderate correlation between the GPAQ and accelerometer on MVPA min/day in self-administered group (*r*_s_ = 0.28 and 0.30, p<0.05) and interviewer-administered group (*r*_s_ = 0.44 and 0.46, p<0.05). In both administration groups combined, the GPAQ and accelerometer were moderately correlated with the accelerometer at moderate-intensity activity level (*r*_s_ = 0.36, p<0.001) and MVPA level (*r*_s_ = 0.39, p<0.001).

**Table 5:** Spearman correlation between the GPAQ and accelerometry-based summary estimates of physical activity level (min/day), according to self- and interviewer-administered groups.

		Self-administered group		Interview-administered group		All	
		(n = 52)		(n = 56)		(n = 108)	
**Total-min physical activity (min/day)**		**r**_**s**_	**95% CI**	**r**_**s**_	**95% CI**	**r**_**s**_	**95% CI**
	MVPA	0.30	0.03–0.53	0.46	0.22–0.65	0.39	0.22–0.54
	Vigorous	0.38	0.12–0.59	0.52	0.30–0.69	0.45	0.29–0.59
	Moderate	0.28	0.01–0.51	0.44	0.20–0.63	0.36	0.18–0.51
**10-min bout physical activity (min/day)**							
	MVPA	0.32	0.05–0.55	0.44	0.20–0.63	0.37	0.20–0.52
	Vigorous	0.35	0.09–0.57	0.43	0.19–0.62	0.39	0.22–0.54
	Moderate	0.29	0.02–0.52	0.42	0.10–0.67	0.20	0.01–0.38

Findings were similar when considering 10-minute bouts of physical activity (Table 5).

Relative to the GPAQ, the Bland-Altman analyses revealed that the accelerometer measured lower daily total MVPA (mean difference; 95% limits of agreement [LOA]: 35.8; -138.7 to 210.4 min/day), vigorous-intensity activity (26.9; -46.2 to 99.9 min/day) and moderate-intensity activity (3.0; -115.0 to 121.0 min/day).

**Fig 2:**
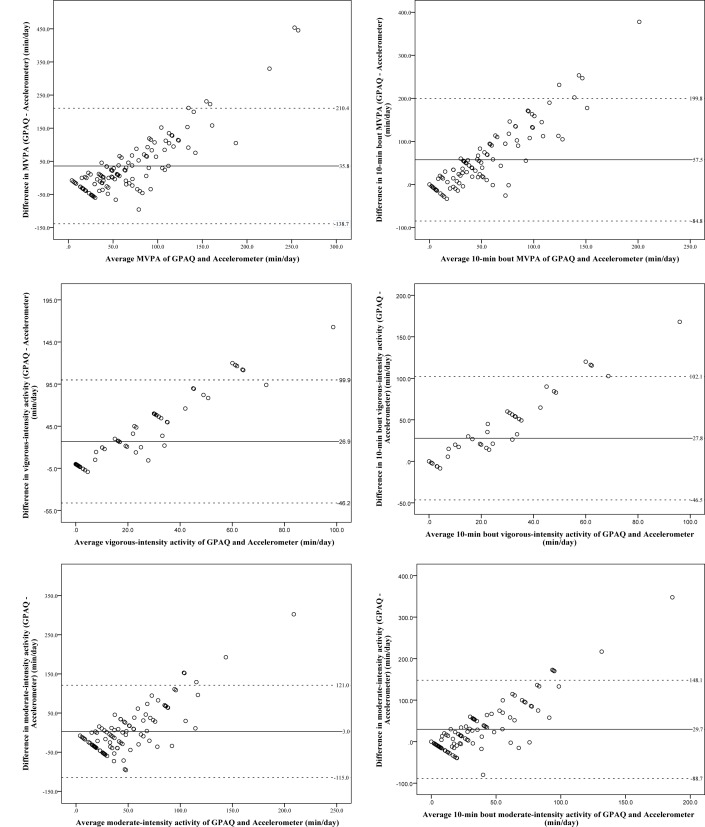
Bland-Altman plots for the agreement of physical activity measurement (min/day) between the GPAQ and accelerometer. Comparisons of total-minute of activity versus a 10-minute bout definition at each of three intensities: MVPA, vigorous-, and moderate- intensity activities for the total sample (n = 108).

When applying the 10-minute bout definition, Bland-Altman plots were similar. Lower accelerometry-derived physical activity was demonstrated as compared to the GPAQ (MVPA: 57.5; -84.8 to 199.8 min/day, vigorous-intensity: 27.8; -46.5 to 102.1 min/day, and moderate-intensity: 29.7; -88.7 to 148.1 min/day) (Fig 2).

The plots illustrate a bias towards overestimation of the MVPA with majority of the points falling above the zero line. The extent of overestimation of vigorous- and moderate-intensity activity level also increased with the duration of activities. Clear upward trends of measurement differences across the range of the measures were apparent in which the measurement differences became greater as the magnitude of reported time increased for each MVPA, vigorous- and moderate-intensity activity.

None of the sensitivity analyses showed dissimilarity across self- and interviewer-administered groups, thus plots were constructed with both groups combined.

## Discussion

To our knowledge, this is the first study to compare the psychometric properties of the GPAQ to measure physical activity between self- and interviewer-administered versions. An aim of this study was to evaluate the self-administered version of the GPAQ with the original interviewer-administered version among a population of literate adults who are fluent in English. The self-administered version performed similarly to the interviewer-administered version, and this would enhance its usability in assessing daily physical activities in population-based surveys by reducing questionnaire administrative burden. Our study presented fair-to-moderate criterion validity of the GPAQ via comparison with the accelerometry-measured physical activity, which is comparable between the two modes. These validity results are consistent with the findings reported by other researchers who validated the interviewer-administered GPAQ [10,15,24]. It was observed that correlations for vigorous-intensity activity were stronger than for moderate intensity, which is consistent with several studies of other physical activity questionnaires [25–27].

Our study also demonstrates that relative to the GPAQ, the accelerometer provided up to almost one hour lower estimates of total MVPA per day, which agrees with earlier findings where an overestimation of self-reported MVPA was observed [28,29]. The Bland-Altman plots demonstrate larger disagreement between the GPAQ and accelerometer at higher levels of MVPA. This pattern indicated overestimation at high activity levels and underestimation at lower activity levels by the GPAQ in our population being studied. The difference between both self- and interviewer-administered approaches seemed to be particularly prominent with regard to vigorous physical activity. This pattern of bias between accelerometry-based physical activity and questionnaires was similar to the findings of other published validation studies [24,30,31]. When accelerometry-based physical activity was determined without the 10-minute bout definition, there would unlikely be nonzero minute of MVPA. As opposed to this, physical activity questionnaires measure physical activity in bouts of 10 minutes, resulting in the inconsistencies between the two measurements. This bias makes the interpretation of questionnaire based findings alone in epidemiological studies problematic.

Our findings are consistent with other research demonstrating stronger correlation in vigorous-intensity activities [26,32]. This may be explained by a more structured nature that is easier to recall. On the other hand, moderate-intensity activities may be both more perceptually and cognitively difficult to recall [33,34]. Identifying ways to improve the accuracy of self-report measures among the population is therefore important in assessing physical activity and trends.

The GPAQ, alongside with other commonly used physical activity questionnaires (e.g. International Physical Activity Questionnaire, IPAQ) were designed to collect physical activity information in accumulated bouts of at least 10-minute per session. Hence, this study considers the implications of using total minutes of activity and 10-minute bouts of activity to determine the accelerometry-derived activity measures. Based upon existing literature, it seems often unclear whether previous validation studies used total activity or 10-minute bouts for the direct comparison with self-report physical activity [24,28]. Several published studies treated the accelerometer activity data in 1-minute bout definition to validate the physical activity questionnaires [35–37]. Our study showed that when accelerometry-derived MVPA in bouts of 10-minute was considered, the overall and vigorous correlations changed only slightly compared to total-minute activity. However, the correlation at moderate-intensity activity estimate dropped substantially from the 1- to 10-minute bout definition. To our knowledge, only one previous study has analyzed the physical activity data using 1-minute and 10-minute bout definitions for the validation of the IPAQ (short form) [29]. They also found that in comparison with the 1-minute bout length, there was a slightly lower correlation between the IPAQ and accelerometry-derived activity when the 10-minute bout definition was employed (*r* = 0.36 and *r* = 0.26, respectively). This seems to confirm the previously highlighted difficulties of accurately recalling moderate-to-vigorous intensity activities, especially activities accumulated in at least 10-minute bouts.

Apart from overestimation by self-report questionnaires, there are issues related to the use of accelerometers that can also contribute towards the discrepancy between both approaches and the observed moderate correlations. For instance, albeit being the most widely used objective tool to measure physical activity, accelerometers are known to underestimate certain activities [38]. Activities like doing housework and cycling which involve only limited movement of the center of mass are poorly detected by accelerometers. In addition, activities such as swimming are often not captured because participants are advised to remove the devices during such activities. This can partly contribute to the discrepancies between self-reported and accelerometry-derived estimates of activity. Although several thresholds and algorithms have been developed for accelerometry-measured physical activity [39–42], there is no consensus on the best method to define physical activity levels and types.

Only few studies seem to have reported the reliability of the GPAQ [43]. In our study, the self-administered version showed comparable reliability with the interviewer-administered version in estimating activity level at each intensity, as well as classification of participants in meeting recommended levels of physical activity. Of note, a lower agreement of the test-retest on total MVPA was demonstrated in the interviewer-administered group. This may be explained by the differences in the domains of physical activities our study population participated in. The reported activity in the transport domain contributed to most total MVPA and showed greater variability as compared to other activities. As the total MVPA was calculated by the sum of vigorous-, moderate-intensity activities of all domains, the observed difference in trend between the Spearman’s correlation and ICC (*r*_*s*_ = 0.61 vs. ICC: 0.28) of the reliability for total MVPA was not unexpected given participants in our study reported engaging more active transport activities and may hence influence the total level of MVPA. In contrast to these observations, the interviewer-administered mode has resulted in better test-retest reliability than self-administered mode in a study of elderly adults’ physical activity [44].

Similar to our observations for validity, reliability was strongest for the assessment of vigorous recreational activities, which is consistent with previous studies [45,46]. This result could be explained by the fact that vigorous activity is predominantly accumulated through recreational and thus likely intentional structured exercise. Participants are able to report such intentional and more well-defined periods of physical activity behavior better than less well-defined ones such as traveling from one place to another or moderate intensity day to day activities.

Our reliability result from the self-administration mode was comparable to Trinh et al.’s [13] in which moderate reliability was presented for total MVPA using the GPAQ. A considerably smaller study by Herrmann et al. reported somewhat better reliability when assessing the GPAQ with an interval of 10 days among US adults aged 43.1 ± 11.4 years [28]. A possible explanation for the differences in physical activity reporting reliability might be related to different study populations.

Variations in correlation coefficients and agreement of test-retest assessments were noted. Nonetheless, different measures suggested that all the GPAQ items provided acceptable reproducibility, which is consistent with results of Lachat et al.’s [47], which showed differences in the test-retest reliability of the IPAQ. The GPAQ showed good reliability in classifying participants’ physical activity levels, which is in line with Herrmann et al.’s [28] study outcomes.

Reliability across all three domains was at least moderate, and the highest reliability was found for recreational activities. In line with our discussion of higher validity and reliability for vigorous intensity activities, this also seems to suggest that the relatively more structured and planned nature of recreational activities may be responsible for these results. However, our findings differ somewhat from those of Bull et al. [15] who reported the highest correlation for the work domain.

The strength of this study is the high compliance with accelerometer wear and adherence with the study protocol. Additionally, our study included a 24-hour accelerometer wear time protocol, which resulted in relatively high wearing durations per day. This might better reflect the physical activity pattern of a participant on a full day than the commonly used approach of focusing on waking time alone. Furthermore, participants were randomly assigned to two administration modes to avoid any potential bias for the comparison between groups. Through this random grouping method, the comparability between the two groups was achieved in relation to their sociodemographic characteristics.

One of the limitations of our study is that the population of our study consisted of mostly full-time working adults and a small number of students from within the university and hospital workplace settings; thus, the results may have limited representativeness for the entire Singaporean population. Also, as the population studied comprised English-speaking adults with more than 50% of them having tertiary education; applicability to other populations cannot be assumed. Second, a bias in estimation of activity which is dependent on the duration of overall activities has been shown by the GPAQ, which introduces some errors. Nonetheless, previous studies have also presented similar findings, thus it is inevitable that the questionnaire are likely to be subject to performance limitations.

## Conclusions

In conclusion, this is among the first studies comparing the reliability and validity of the internationally widely used GPAQ considering two different modes of administration. Moreover, a 24-hour wear time protocol was employed and considered two different scenarios for accelerometer data processing. Our findings show that both interviewer- and self-administered modes of the GPAQ are comparable. Evidence for criterion validity was shown with fair-to-moderate correlation coefficients, of which the self-administration can be used in population-based studies.

However, there was potential bias in estimation of activity differing at different intensities by the GPAQ. It should be noted that the pattern of over- and underestimation from the GPAQ is unpredictable; and these responses are dependent on the overall physical activity. Therefore, the use of GPAQ as a tool for investigation of adult physical activity patterns should be undertaken with caution.

Future epidemiological studies could incorporate the GPAQ with a good understanding of various types and domains in which physical activity is carried out; together with objectively measured physical activity that provide a more accurate measure of overall activity levels and at various activity intensity levels.
